# Army Legislative Committee Appeal

**Published:** 1900-04

**Authors:** Rush S. Huidekoper

**Affiliations:** Secretary; Washington


### A. V. M. A.

### ARMY LEGISLATIVE COMMITTEE APPEAL.

### Brief of Facts in Support of Senator Kenney's Amendment to the Army Appropriation Bill.[1]

Senator Kenney's amendment to the army appropriation bill provides for the establishment of an organized veterinary service, with reputable officers, whose education and competency are insured by rigid examination; to replace the present system of employment of an unorganized class of individual civilians, with no rank, no future, no responsibility, and no official position.

The United States Army is absolutely deficient in a proper veterinary service, which exists in the army of every other civilized country in the world. The present system in the United States Army has less method in it than that found needful in any large contractor's stable or stock farm in the country.

*Economy.* The entire cost under the amendment is only six thousand five hundred dollars over the present cost of civilian employés under the present system, and will be more than compensated for by the economy resulting from an organized veterinary corps.

*Efficiency.* The establishment of an organized Veterinary Service:

To give proper instruction in hippopathology, inspection of forage, stable hygiene, and farriery, to officers, cadets, non-commissioned officers and farriers, at our military schools and large posts.

To furnish expert veterinarians for service as a member on boards of purchase of remounts.

To furnish qualified Inspectors of live stock and dressed meat for our soldiers' rations (there is not a qualified meat-inspection service in the U. S. Army).

Is needed, both on the ground of economy, for the efficiency of the Army, and for the health of our soldiers.

The letters, copies of which have been furnished, from Major-General Merritt, Major-General Brooke, Major-General Wilson, and numerous others of the higher officers of the Army, indorse such an organization.

The establishment of such an organization has had the approval of President William McKinley.

[1] Calendar No. 968, H. R. 8582.

The General commanding the Army, Major-General Miles, and the Quartermaster-General, Brig.-General Ludington, strongly approve and favor the amendment.

The personal consideration of the merits of this amendment and the individual vote of each Senator are respectfully requested.

RUSH S. HUIDEKOPER,

Secretary.

WASHINGTON, April 25, 1900.

